# Hybrid Multidelay PCASL Acquired With Time‐Encoded and Variable‐TR Schemes for the Assessment of Cerebral Perfusion in Moyamoya Disease

**DOI:** 10.1002/nbm.70069

**Published:** 2025-05-23

**Authors:** Osamu Togao, Makoto Obara, Koji Yamashita, Kazufumi Kikuchi, Tatsuhiro Wada, Chiaki Tokunaga, Ryoji Mikayama, Koichi Arimura, Ataru Nishimura, Shota Ishida, Lena Václavů, Matthias J. P. van Osch, Kim van de Ven, Marc van Cauteren, Kousei Ishigami

**Affiliations:** ^1^ Department of Molecular Imaging and Diagnosis, Graduate School of Medical Sciences Kyushu University Fukuoka Japan; ^2^ Philips Japan Tokyo Japan; ^3^ Department of Clinical Radiology, Graduate School of Medical Sciences Kyushu University Fukuoka Japan; ^4^ Division of Radiology, Department of Medical Technology Kyushu University Hospital Fukuoka Japan; ^5^ Department of Neurosurgery, Graduate School of Medical Sciences Kyushu University Fukuoka Japan; ^6^ Department of Radiological Technology, Faculty of Medical Sciences Kyoto College of Medical Science Kyoto Japan; ^7^ C.J. Gorter MRI Center, Department of Radiology Leiden University Medical Center Leiden the Netherlands; ^8^ Philips Healthcare Best the Netherlands

**Keywords:** arterial spin labeling, cerebral perfusion, Moyamoya disease

## Abstract

Multidelay arterial spin labeling (ASL) MRI requires balancing temporal information with signal‐to‐noise ratio (SNR). A hybrid scheme combines the high SNR of time‐encoded pseudo‐continuous ASL (PCASL) with the temporal flexibility of variable‐TR PCASL. The purpose of the present study was to assess the SNR and quantitative performance of the hybrid scheme by comparing it to time‐encoded and variable‐TR schemes for evaluating cerebral perfusion in patients with moyamoya disease. Twenty patients with moyamoya disease (8 men and 12 women, 27.6 ± 22.7 years) were scanned with 3 T MRI system. Variable‐TR PCASL with 12 delays (vTR12), time‐encoded PCASL with seven delays (TEnc7), and hybrid PCASL with 12 (4 × 3) delays (Hyb12) were obtained. Hyb12 and vTR12 were configured with nearly identical label duration (LD) and postlabeling delay (PLD) settings. For TEnc7, it was balanced so that the maximum LD + PLD time is the same. Time‐corrected SNR (SNR_t_), cerebral blood flow (CBF), and arterial transit time (ATT) were measured in vascular territories within gray matter. The three parameters were compared using the Friedman's matched test, followed by the Dunn's multiple comparison test. Correlations were evaluated with Pearson's correlation, and agreement was assessed by Bland–Altman plot analysis and intraclass correlation coefficient (ICC). The SNR_t_ of Hyb12 (4.67 ± 1.97) was significantly higher than that of vTR12 (3.27 ± 1.37, *p* < 0.0001) and TEnc7 (3.52 ± 1.32, *p* < 0.0001). The CBF of Hyb12 (49.9 ± 13.7 mL/100 g/min) was significantly lower than that of vTR12 (54.9 ± 15.4 mL/100 g/min, *p* < 0.0001) and TEnc7 (57.5 ± 15.9 mL/100 g/min, *p* < 0.0001). The ATT of Hyb12 (1240 ± 430 ms) was significantly shorter than that of vTR12 (1474 ± 419 ms, *p* < 0.0001) and TEnc7 (1402 ± 333 ms, *p* < 0.0001). CBF and ATT measurements from Hyb12 showed strong correlations and good agreement with the other two schemes. The hybrid scheme offers higher SNR_t_ than both time‐encoded and variable‐TR schemes, which may improve the accuracy of cerebral perfusion assessment in patients with cerebral artery occlusive diseases, where delayed blood flow is a concern.

## Graphical abstract

Schematic drawings of the hybrid PCASL, consisting of four time‐encoded schemes with different timing settings, are shown. The entire LD/PLD is rearranged to show bolus dynamics. Actual multidelay images obtained with the hybrid scheme in a patient with moyamoya disease, along with his CBF and ATT maps, are shown.

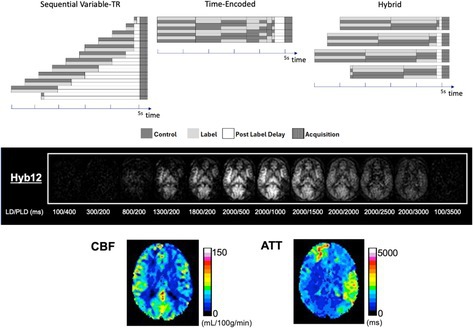

**Abbreviations**
ASL: arterial spin labeling
ATT: arterial transit time
CBF: cerebral blood flow
Hyb12: hybrid PCASL with 12 delays
ICC: intraclass correlation coefficient
LD: labeling duration
LLLD‐PCASL: long‐delayed pseudocontinuous arterial spin labeling
PCASL: pseudocontinuous arterial spin labeling
PET: positron emission tomography
PLD: postlabeling delay
SNR: signal‐to‐noise ratio
SPECT: single photon emission computed tomography (SPECT)
TEnc7: time‐encoded PCASL with seven delays
SNR_t_
: time‐corrected signal‐to‐noise ratio
vTR12: variable‐TR PCASL with 12 delays

## 1 Introduction

Moyamoya disease is a cerebrovascular occlusive disorder primarily affecting East Asians, including the Japanese population [1]. This condition is characterized by progressive stenosis and occlusion of the bilateral internal carotid arteries and the circle of Willis, as observed on cerebral angiography, along with the development of a distinctive network of blood vessels at the base of the brain known as moyamoya vessels.

Revascularization surgery for moyamoya disease patients is indicated based on the presence of ischemic symptoms and the status of cerebral perfusion. Clinically, cerebral perfusion in these patients is evaluated using single photon emission computed tomography (SPECT) or positron emission tomography (PET). It has been shown that patients with preoperative impairment of cerebral blood flow (CBF) experience improvement after revascularization [2, 3]. The Japanese guideline for diagnosing and treating moyamoya disease recommends the use of SPECT or PET for evaluating cerebral perfusion, diagnosing cerebral ischemia, assessing severity, determining surgical indications, and managing perioperative care in ischemic moyamoya disease (recommendation level B) [4, 5]. However, these nuclear medicine techniques are available at only a limited number of facilities and carry risks associated with radiation exposure.

Recently, arterial spin labeling (ASL) MRI has become more prevalent in clinical practice for evaluating cerebral perfusion [6]. Nevertheless, there is no established method to acquire ASL that provides accurate CBF measurements in moyamoya disease. To assess the delayed blood flow seen in this disease, imaging with a long delay and sufficient signal‐to‐noise ratio (SNR) is required. Single‐phase ASL quantification of CBF for conventional clinical use is unreliable in tissues with prolonged arterial transit time (ATT), as observed in this disease. Multidelay pseudocontinuous ASL (PCASL) using a time‐encoded scheme has been reported for imaging in moyamoya disease [7]. The time‐encoded scheme divides the PCASL labeling module into a series of blocks, encoded as either label or control in various combinations across the acquisition series, based on a Hadamard encoding matrix [8, 9, 10]. Due to its inherent noise‐averaging effects, the time‐encoded scheme provides a high SNR per unit of time [8, 10]. However, the labeling duration (LD) and postlabeling delay (PLD) settings for each subbolus are interdependent, resulting in low flexibility in timing parameter settings. Whereas LD and PLD are particularly important parameters for the measurement of ATT and CBF at sufficient SNR, the time‐encoded scheme does not allow for the individual optimization of these conditions for each subbolus as subboli cannot overlap. In addition, increasing the number of subboli improves temporal resolution but shortens the LD of each subbolus, which reduces SNR and consequently the accuracy of ATT and CBF quantification.

Another time‐efficient approach is the “sequential multidelay combined with a variable repetition time (variable‐TR) scheme” [11, 12]. Multidelay acquisition, with dynamic changes in LD and PLD, minimizes TR for each delay based on their specific combination to enhance time efficiency. This scheme allows any LD or PLD to be set without restriction. It has been previously reported that variable‐TR PCASL with an optimized background suppression scheme is useful for assessing cerebral perfusion in moyamoya disease [13]. Moreover, the quantitative CBF measured by the variable‐TR scheme showed a good correlation with that measured by iodine‐123‐N‐isopropyl‐p‐iodoamphetamine SPECT [13]. However, a disadvantage of variable‐TR PCASL is its lower SNR_t_ compared to time‐encoded PCASL.

Recently, a hybrid scheme combining the concepts of time‐encoded and variable‐TR schemes has been proposed, which is expected to combine both the high SNR_t_ of time‐encoded PCASL with the temporal flexibility of variable‐TR PCASL while maintaining time efficiency [14, 15, 16]. The aim of this study was to investigate the quantitative performance of the hybrid scheme by comparing it to the two major multidelay PCASL techniques (time‐encoded and variable TR) in assessing cerebral perfusion in moyamoya disease.

## 2 Materials and Methods

This investigation was approved by the Institutional Review Board of Kyushu University Hospital (No. 22049‐00). Subjects were imaged after obtaining informed consent. Three of the authors (M.O., M.V.C., and K.v.d.V) are employees of Philips Healthcare and provided technical support for the sequence development but were not involved in the study design or interpretation of the data. The institutional authors, who were not Philips Healthcare employees, were responsible for all handling of data.

### 2.1 Patients

Since April 2022, our hospital's routine MR imaging protocol for moyamoya disease has included the multidelay ASL sequences. Herein, we analyzed the imaging data of 20 consecutive inpatients with moyamoya disease identified between April 2022 and November 2023. All patients had been diagnosed with moyamoya disease by cerebral angiography. The exclusion criteria for this study were as follows: Major artifacts due to motion, labeling failure, or other causes were not observed in any of the images (*n* = 0).

### 2.2 MRI

**FIGURE 1:**
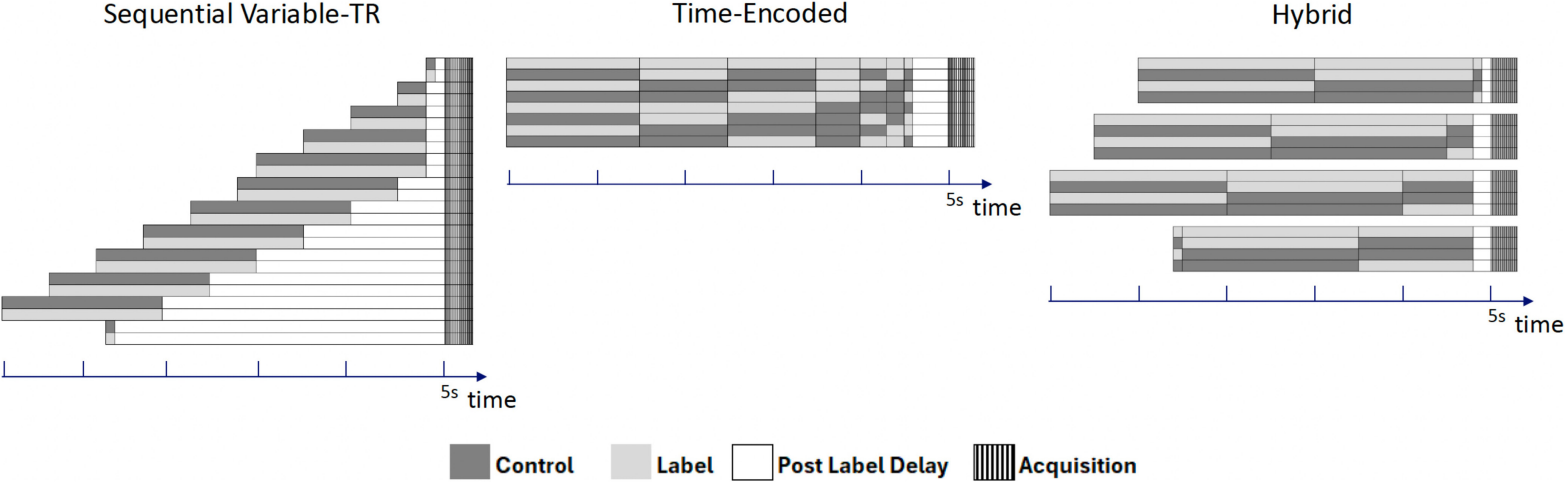
Schematic representation of the three ASL acquisition schemes: sequential variable‐TR, time‐encoded, and hybrid. The diagram illustrates the timing of control, labeling, postlabeling delay, and acquisition for each scheme. The sequential variable‐TR scheme dynamically adjusts the label duration (LD) and postlabeling delay (PLD), minimizing the TR for each delay based on their specific combination to enhance time efficiency. This scheme allows for the flexible selection of any LD or PLD without restriction. The time‐encoded scheme divides the PCASL labeling module into seven blocks, which are encoded as either label or control in various combinations across the acquisition series based on a Hadamard encoding matrix. The Hybrid scheme consists of four time‐encoded acquisitions with different timing settings. The label duration is subdivided into three blocks for each of the three acquisitions. This approach allows for relatively flexible timing settings for labeling and postlabeling delay.

A 3T scanner (Ingenia Elition, Philips) with a 32‐channel head coil was used. The schematic drawings of the variable‐TR, time‐encoded, and hybrid schemes are shown Figure 1. The hybrid scheme consists of four Hadamard encodings with different timing setting, resulting in 12 measurements in total. It can be seen that the hybrid approach offers flexibility in choosing short or long LDs in combination with short or long PLDs. For this study, the final LD/PLD combination of 100/3500 ms is a superdelay phase obtained for noise measurements.

The MRI‐protocol included three multidelay PLD PCASL variants: variable‐TR scheme with 12 delays (vTR12), time‐encoded scheme with seven delays (TEnc7), and hybrid scheme with 4 × 3 delays (Hyb12). The actual LDs, PLDs, are summarized in Table 1. For vTR12, the TR corresponding to LD/PLD is also included in the list. Imaging parameters are summarized in Table 2. For Hyb12, the TR for each Hadamard encoding is listed in the order of execution. In the all ASL sequences, the same 3D gradient‐ and spin‐echo sequence covering a 120‐mm‐thick slab was used for all three ASL sequences. The field of view was 256 × 256 mm, the matrix size was 64 × 64, and the number of averages was two for all sequences. The total scan time was 2 min 49 s for Hyb12, 3 min 0 s for vTR12, and 1 min 30 s for TEnc7. Improved motion‐sensitized driven‐equilibrium was employed for vascular signal suppression with a preparation echo time of 12.5 ms and velocity encoding of 5 cm/s in all directions [17]. M0 images were acquired, and background suppression was applied to all schemes. All scans were performed in the same session. In the vTR12 and TEnc7 schemes, ASL images were created by decoding the image data encoded using Hadamard coding via the Inverse Hadamard Transform, whereas in the vTR scheme, ASL images were generated by subtracting the labeled images from the control images.

**TABLE 1:** Label duration and postlabeling delay combinations and TR in the vTR scheme.

Hyb12	LD (ms)	100	300	800	1300	1800	2000	2000	2000	2000	2000	2000	100
	PLD (ms)	100	200	200	200	200	500	1000	1500	2000	2500	3000	3500
vTR12	LD (ms)	100	300	800	1300	1800	2000	2000	2000	2000	2000	2000	100
	PLD (ms)	400	200	200	200	200	500	1000	1500	2000	2500	3000	3500
	TR (ms)	1550	1550	2050	2550	3050	3550	4050	4550	5050	5550	6050	4650
TEnc7	LD (ms)	100	200	300	500	1000	1000	1500					
	PLD (ms)	400	500	700	1000	1500	2500	3500					

Abbreviations: Hyb12, hybrid scheme with 12 delays; LD, label duration; PLD, postlabeling delay; TEnc7, time‐encoded scheme with 7 delays; vTR12, variable repetition time scheme with 12 delays.

**TABLE 2:** Imaging acquisition parameters.

Scheme	Hyb12	vTR12	TEnc7
Sequence	3D‐GRASE	3D‐GRASE	3D‐GRASE
TR (ms)	5050, 5550, 6050, 4650	1550–6050^a^	6050
TE (ms)	15	15	15
Flip angle (degree)	90	90	90
TSE, EPI factor	16, 15	16, 15	16, 15
Voxel size (mm)	4.0 * 4.0 * 8.0	4.0 * 4.0 * 8.0	4.0 * 4.0 * 8.0
Slab thickness (mm)	120	120	120
Vascular crusher type	iMSDE	iMSDE	iMSDE
VENC (cm/s)	5 (each of the three axes)	5 (each of the three axes)	5 (each of the three axes)
Scan time	2 min 49 s	3 min 00 s	1 min 30 s

Abbreviations: EPI, echo planar imaging; GRASE, gradient and spin echo; Hyb12, hybrid scheme with 12 delays; iMSDE, improved motion‐sensitized driven‐equilibrium; TE, echo time; TEnc7, time‐encoded scheme with 7 delays; TR, repetition time; TSE; turbo spin echo; VENC, velocity encoding; vTR12, variable repetition time scheme with 12 delays.

^a^ TR for each LD/PLD is shown in Table 1.

Three‐dimensional T1‐weighted anatomical images were also collected with the following sequence parameters: sequence, inversion‐recovery prepared 3D segmented field echo; TR/echo‐time (TE), 7.8/3.6 ms, inversion time, 974 ms; flip angle, 8°; turbo factor, 240; voxel size, 1.0 × 1.0 × 1.0 mm (190 partitions); 3D slab thickness, 190 mm; orientation, sagittal; compressed‐sensing combined sensitivity encoding factor, 5.0; and total scan time, 2 min 11 s.

### 2.3 Image Analysis

#### 2.3.1 SNR, ATT, and CBF Quantification

In the multidelay series, the ASL maps with the highest three signals along the time axis (defined as the mean ASL‐signal in the gray matter for a certain LD/PLD combination) were selected and subsequently averaged voxel by voxel. In the super‐delay phase (LD/PLD: 100/3500 ms) of vTR12 and Hyb12, and the initial phase (LD/PLD: 100/400 ms) of TEnc, the labeling time is short, and the delay is either extremely long or short, resulting in minimal labeled blood flow in the brain tissue. Therefore, these scans can be considered noise scans. Based on this, the standard deviation of the residual signal in the gray matter across the entire volume was calculated in the super‐delay phase for vTR12 and Hyb12, and in the initial phase for TEnc7, to estimate the noise level. The SNR was calculated voxel by voxel by dividing the average of the three highest ASL signal values by the standard deviation obtained from the noise scan. Time‐corrected SNR (SNR_t_) was defined as the SNR divided by the square root of the scan time for each sequence, which was used for fair comparison of SNR. CBF and ATT maps were generated from all dynamic ASL data, excluding the super‐delay image. To calculate CBF and ATT, the general kinetic model of Buxton et al. [18] was fitted to the following equation using in‐house nonlinear fitting coded in Python 3.7 to the following equation:

$$\begin{array}{c} S_{\mathrm{ASL}}=2\cdot M_{0}\cdot \mathrm{CBF}\cdot \alpha_{\text{label}}\cdot \alpha_{\mathrm{BGS}}\cdot T_{1t}\cdot e^{-\frac{\mathrm{ATT}}{T_{1a}}}\\ \;\cdot \left(e^{\frac{-\max\left({\mathrm{PLD}}_{i}-\mathrm{ATT},0\right)}{T_{1t}}}-e^{\frac{-\max\left({\mathrm{LD}}_{i}+{\mathrm{PLD}}_{i}-\mathrm{ATT},0\right)}{T_{1t}}}\right)/\lambda . \end{array}$$

Here, *S*_*ASL*_ is the signal intensity on an ASL image with a certain LD/PLD combination; *M*_0_ is the equilibrium magnetization; *α*_*label*_ is the labeling efficiency; *α*_*BGS*_ is the efficiency of background suppression; *T*_1*t*_ is the tissue T1; *T*_1*a*_ is the blood T1; and *λ* is the tissue‐blood partition coefficient. Literature values were used for the following parameters: *α*_*label*_ = 0.85 [6]; *α*_*BGS*_ = 0.8 [19]; *T*_1*t*_ = 1.5 s [11]; *T*_1*a*_ = 1.66 s [11]; and *λ* = 0.9 [6].

Gray matter probability was calculated from the T1‐weighted images using SPM12 (The Wellcome Trust Centre for Neuroimaging, https://www.fil.ion.ucl.ac.uk/spm/software/spm12/) [20] and then coregistered and downsampled to PCASL image resolution, and the gray matter mask was created by applying a threshold of 0.5. The same gray matter mask was applied to the perfusion parameter maps across all schemes. All maps were normalized to the Montreal Neurological Institute space template using SPM12. The processing pipeline was carefully inspected and confirmed to function properly for pediatric patients, including a 1‐year‐old infant. No significant segmentation or registration issues were observed. Volumes of interest were obtained from a vascular territory atlas template [21]: The measurements were performed in the anterior cerebral artery, middle cerebral artery, and posterior cerebral artery territories—with subdivision into proximal, middle, and distal regions—for both left and right hemisphere [21]. This resulted in 18 volumes of interest in one patient.

### 2.4 Statistical Analyses

All values are expressed as means ± standard deviations. Averaged SNR_t_, CBF, and ATT in the gray matter volumes of interest were compared among the three schemes using Friedman matched test followed by Dunn's multiple comparison test. Correlations between CBF or ATT obtained by the two of the three methods were evaluated by Pearson correlation. The agreement between them was evaluated using Bland–Altman plot analysis and intraclass correlation coefficient (ICC). An ICC of less than 0.50 is considered poor, between 0.50 and 0.75 is considered moderate, between 0.75 and 0.90 is considered good, and greater than 0.90 is considered excellent [22]. Statistical analyses were performed with commercially available software packages (Prism 10.2.3: GraphPad Software, San Diego, CA; MedCalc Version 22.023: MedCalc Software, Ostend, Belgium). *p* values of < 0.05 were considered significant.

## 3 Results

A total of 20 patients (8 men and 12 women) with moyamoya disease were included in the present study. The mean age was 27.6 ± 22.7 years (range 1–72 years). Five of the 20 patients had undergone cerebral bypass surgery in the past.

**FIGURE 2:**
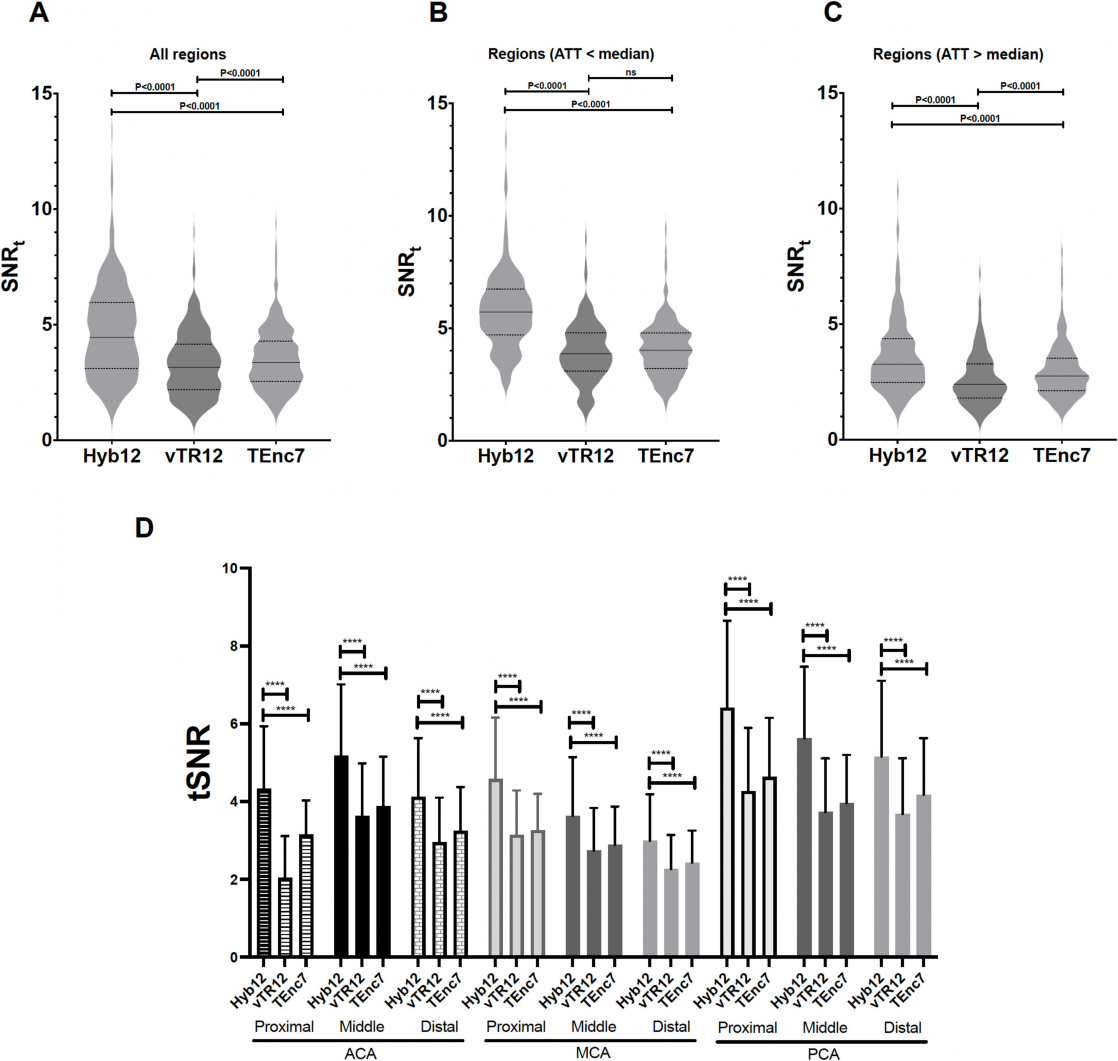
The comparisons of SNR_t_. (A) In all regions, the SNR_t_ of Hyb12 was significantly higher than those of the other two schemes. The SNR_t_ of TEnc was significantly higher than that of vTR12. (B) In regions where ATT was shorter than the median, the SNR_t_ of Hyb12 was significantly higher than that of vTR12 and TEnc7. (C) In regions where ATT was longer than the median, the SNR_t_ of Hyb12 was significantly higher than those of the other two schemes. The SNR_t_ of TEnc was significantly higher than that of vTR12. (D) In all vascular territories, the SNR_t_ of Hyb12 was significantly higher than that of vTR12 and TEnc7, whereas no differences were observed between vTR12 and TEnc7.

Figure 2 demonstrates the comparisons of SNR_t_ measured in the volumes of interest among the three schemes. In all regions (Figure 2A), the SNR_t_ of Hyb12 (4.67 ± 1.97) was significantly higher than that of vTR12 (3.27 ± 1.37, *p* < 0.0001) and TEnc7 (3.52 ± 1.32, *p* < 0.0001). The SNR_t_ of TEnc7 was significantly higher than that of vTR12 (*p* < 0.0001). To account for the effect of ATT, separate analyses were performed for regions with ATT shorter and longer than median. In regions where ATT was shorter than the median (Figure 2B), the SNR_t_ of Hyb12 (5.77 ± 1.70) was significantly higher than that of vTR12 (3.93 ± 1.29, *p* < 0.0001) and TEnc7 (4.08 ± 1.22, *p* < 0.0001). In regions where ATT was longer than the median (Figure 2C), the SNR_t_ of Hyb12 (3.58 ± 1.56) was significantly higher than that of vTR12 (2.61 ± 1.10, *p* < 0.0001) and TEnc7 (2.95 ± 1.18, *p* < 0.0001). The SNR_t_ of TEnc7 was significantly higher than that of vTR12 (*p* < 0.0001). In all vascular territories, averaged across both hemispheres, the SNR_t_ of Hyb12 was significantly higher than that of vTR12 and TEnc7, whereas no differences were observed between vTR12 and TEnc7 (Figure 2D).

**FIGURE 3:**
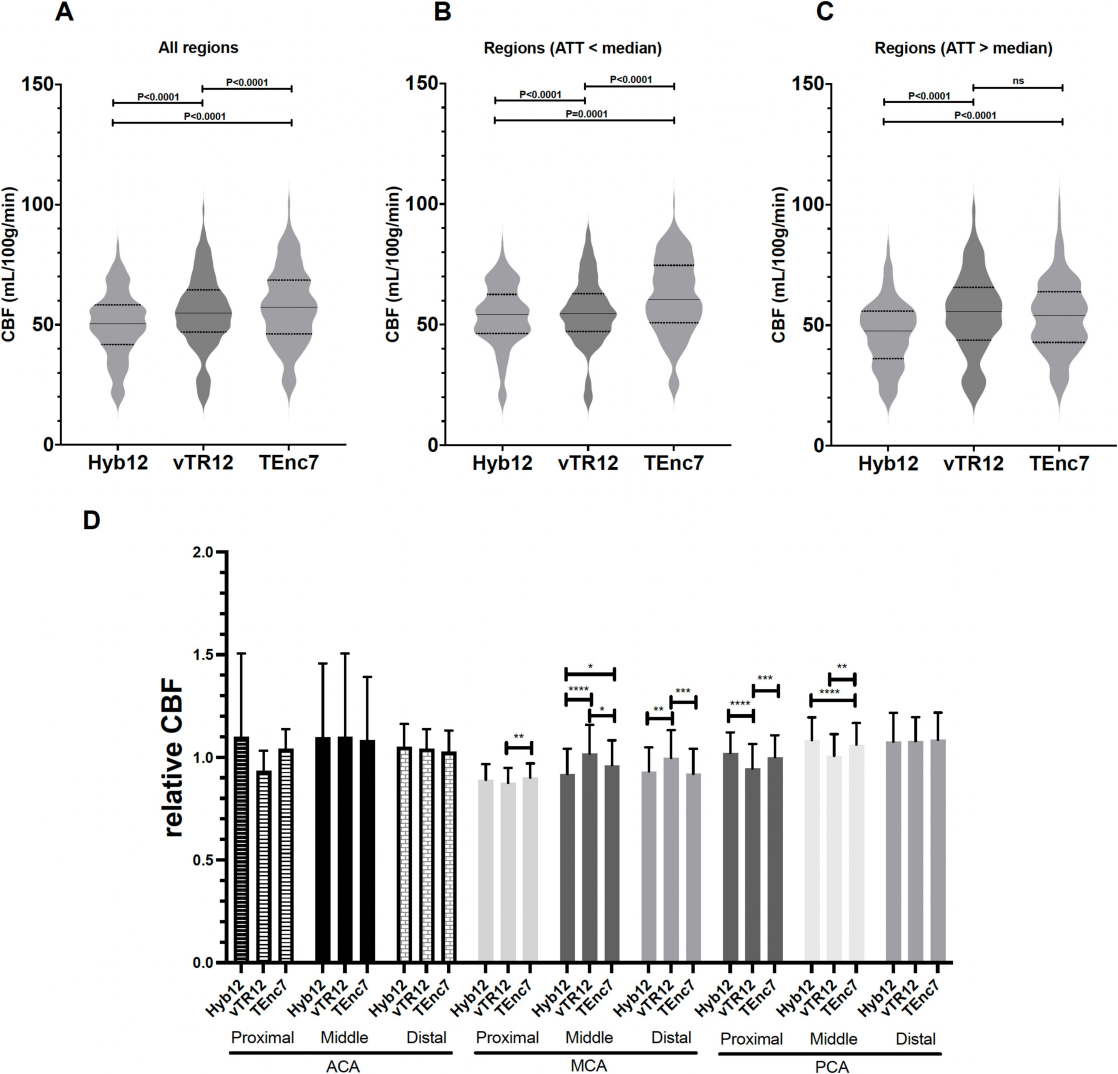
The comparisons of CBF. (A) In all regions, the CBF of Hyb12 was significantly lower than that of vTR12 and TEnc7. The CBF of TEnc was significantly higher than that of vTR12. (B) In regions where ATT was shorter than the median, the CBF of Hyb12 was significantly lower than that of vTR12 and TEnc7. The CBF of TEnc was significantly higher than that of vTR12. (C) In regions where ATT was longer than the median, the CBF of Hyb12 was significantly lower than that of vTR12and TEnc7. (D) The relative CBF, obtained by dividing the value for each vascular region by the mean value of all 18 regions. Significant differences in relative CBF among the schemes are observed across all portions of the middle cerebral artery territories, as well as in the proximal and middle portions of the posterior cerebral artery territories.

Figure 3 demonstrates the comparisons of CBF measured in the volumes of interest among the three schemes. In all regions (Figure 3A), the CBF of Hyb12 (49.9 ± 13.7 mL/100 g/min) was significantly lower than that of vTR12 (54.9 ± 15.4 mL/100 g/min, *p* < 0.0001) and TEnc7 (57.5 ± 15.9 mL/100 g/min, *p* < 0.0001). The CBF of TEnc7 was significantly higher than that of vTR12 (*p* < 0.0001). In regions where ATT was shorter than the median (Figure 3B), the CBF of Hyb12 (53.5 ± 12.9 mL/100 g/min) was significantly lower than that of vTR12 (55.1 ± 14.0 mL/100 g/min, *p* = 0.0047) and TEnc7 (61.4 ± 15.5 mL/100 g/min, *p* < 0.0001). The CBF of TEnc7 was significantly higher than that of vTR12. In regions where ATT was longer than the median (Figure 3C), the CBF of Hyb12 (46.3 ± 13.6 mL/100 g/min) was significantly lower than that of vTR12 (54.8 ± 16.8 mL/100 g/min, *p* < 0.0001) and TEnc7 (53.6 ± 15.3 mL/100 g/min, *p* < 0.0001). The relative CBF, obtained by dividing the value for each vascular region by the mean value of all 18 regions, is shown in Figure 3D. Significant differences in relative CBF among the schemes are observed across all portions of the middle cerebral artery territories, as well as in the proximal and middle portions of the posterior cerebral artery territories. The relative CBF obtained with vTR12 was higher than that of the other two schemes in the middle and distal portions of the middle cerebral artery territories and lower in the proximal and middle portions of the posterior cerebral artery territories.

**FIGURE 4:**
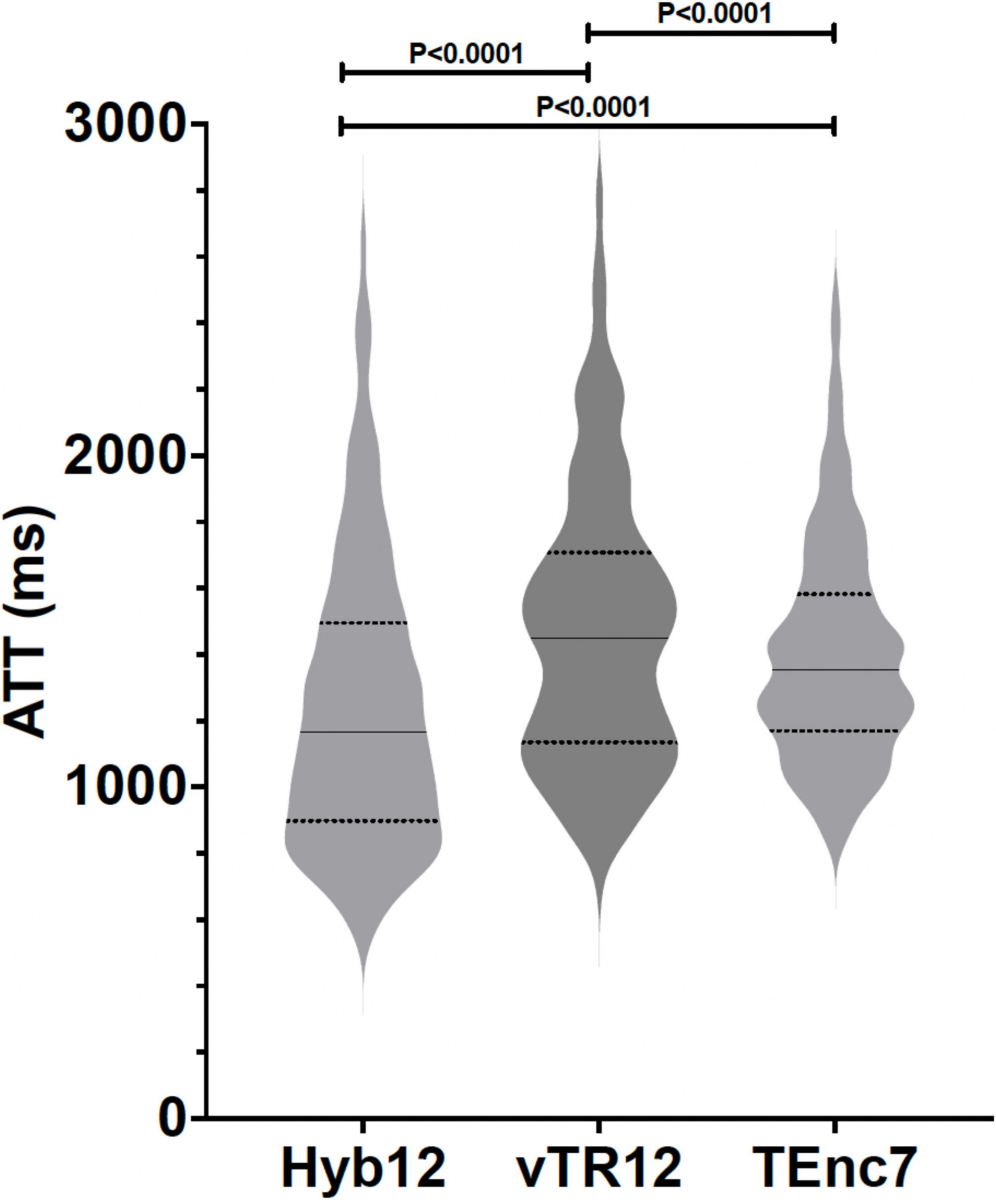
The comparisons of ATT. The ATT of Hyb12 was significantly shorter than that of those of the other two schemes. The ATT of vTR12 was significantly longer than that of TEnc7.

Figure 4 demonstrates the comparisons of ATT measured in all volumes of interest among the three schemes. The ATT of Hyb12 (1240 ± 430 ms) was significantly shorter than that of vTR12 (1474 ± 419 ms, *p* < 0.0001) and TEnc7 (1402 ± 333 ms, *p* < 0.0001). The ATT of vTR12 was significantly longer than that of TEnc7 (*p* < 0.0001).

**FIGURE 5:**
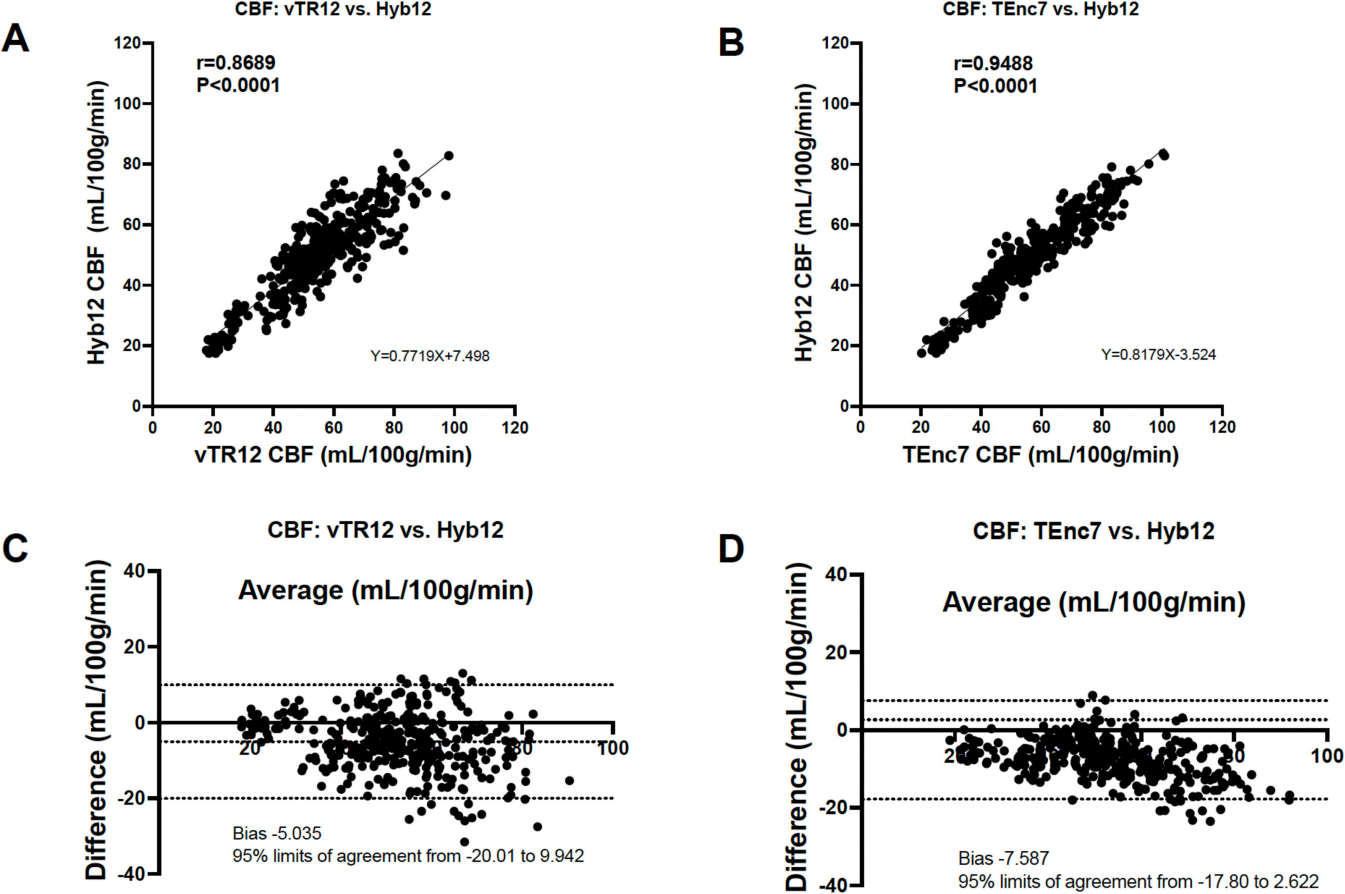
The correlations and Bland–Altman plots for CBF. The CBF measured with Hyb12 shows significant correlations with those measured with vTR12 (A) and TEnc7 (B). The Bland–Altman plot analysis for CBF shows a small fixed bias without significant proportional bias for both comparisons (C, D).

**FIGURE 6:**
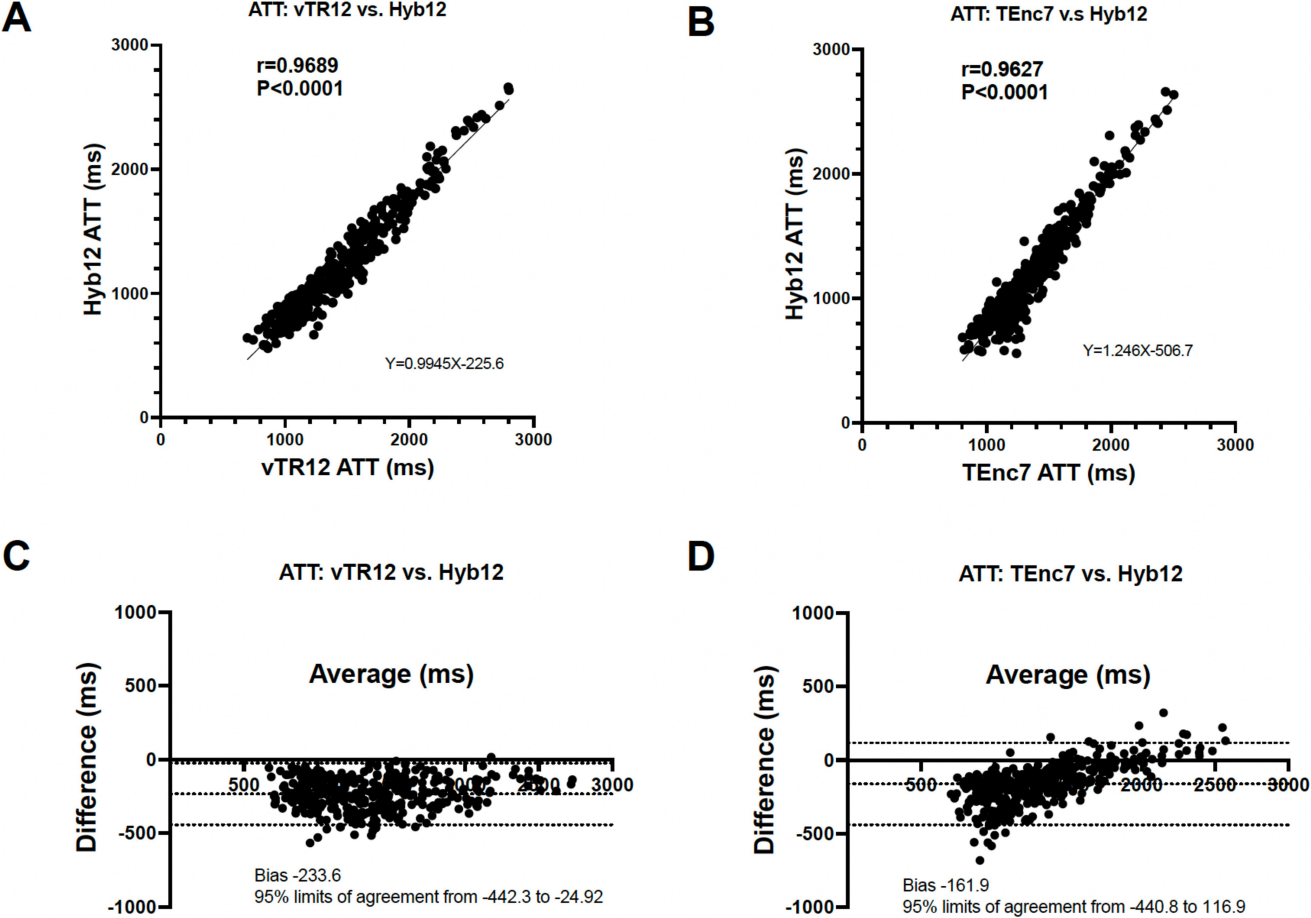
The correlations and Bland–Altman plots for ATT. The ATT measured with Hyb12 shows significant correlations with those measured with vTR12 (A) and TEnc7 (B). The Bland–Altman plot analysis for ATT between Hyb12 and vTR12 shows a relatively large fixed bias without proportional bias (C). The Bland–Altman plot analysis for ATT between Hyb12 and TEnc7 shows a relatively large fixed bias with proportional bias (D).

**FIGURE 7:**
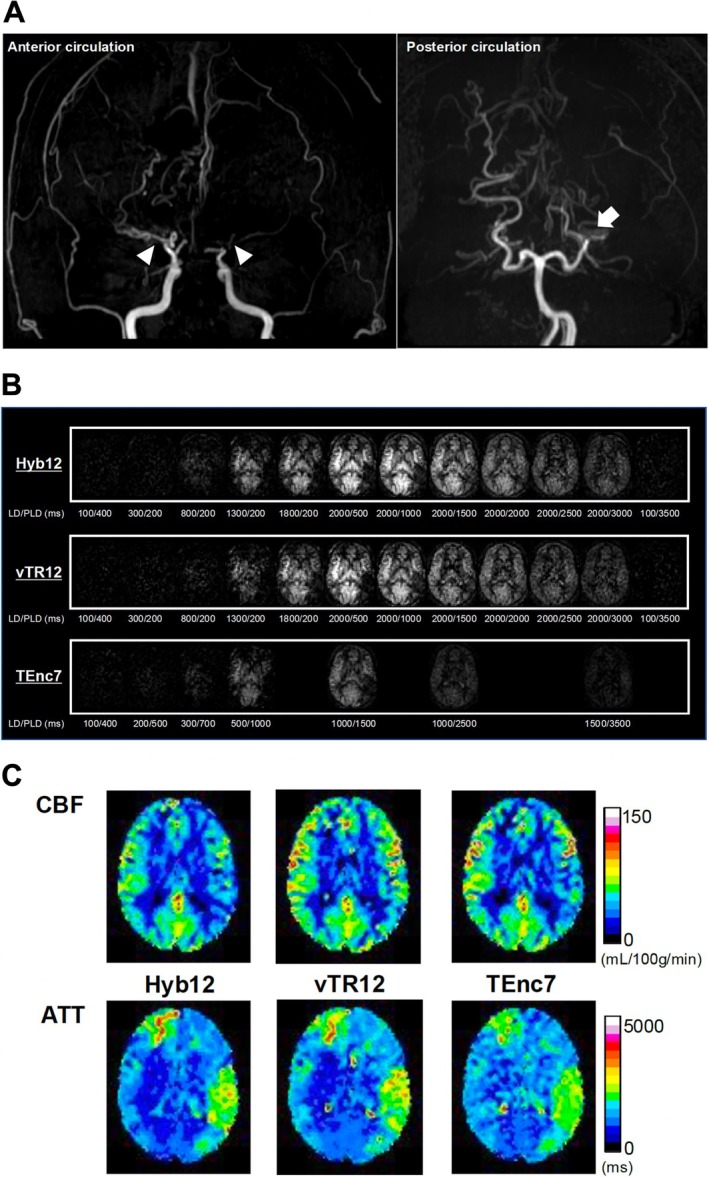
Male in his 40s with moyamoya disease. (A) 3D time‐of‐flight MR angiography shows occlusions of the bilateral internal carotid arteries (arrowheads) and left posterior cerebral artery (arrow). (B) Hyb12 provides images with higher SNR than vTR12 and TEnc7. All images are shown with the same signal scale for comparison, (C) CBF maps (top row) show that overall CBF is preserved throughout both cerebral hemispheres in all schemes. However, in Hyb12, the CBF appears slightly reduced in the left middle cerebral artery territory and watershed areas. The overall CBF observed with Hyb12 is slightly lower than that obtained with the other two schemes. ATT maps (bottom row) show that ATT is prolonged in the right anterior cerebral artery and posterior left posterior middle cerebral artery territories in all three schemes. The distinction between regions with short and long ATT is most clearly depicted in Hyb12, whereas in TEnc7, this distinction is less pronounced.

**FIGURE 8:**
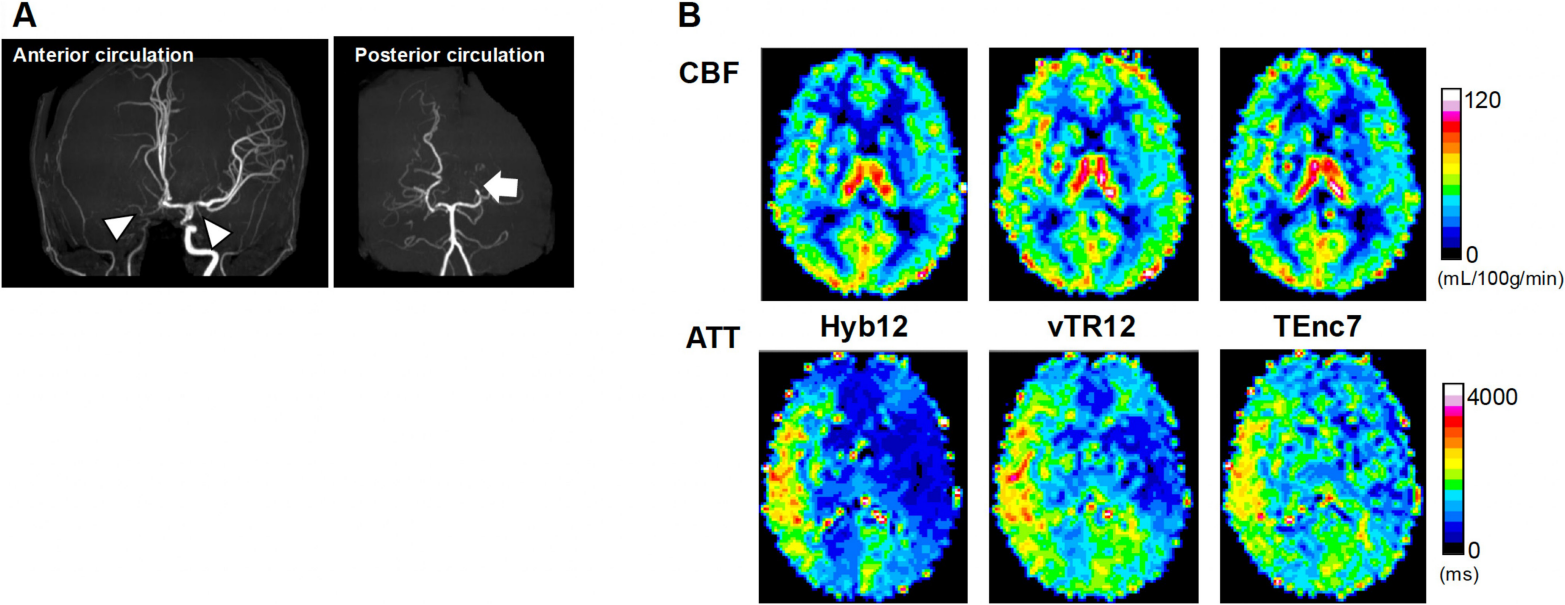
Female in her 40s with moyamoya disease. (A) Three‐dimensional time‐of‐flight MR angiography shows an occlusion of the right internal carotid artery and stenosis of the left internal carotid artery (arrowheads) and the left posterior cerebral artery (arrow). (B) CBF maps (top row) show that overall CBF is preserved throughout both cerebral hemispheres in all schemes. The overall CBF observed with Hyb12 is slightly lower than that obtained with the other two schemes. ATT maps (bottom row) show that ATT is prolonged in the right middle cerebral artery and left posterior cerebral artery territories in all three schemes. The distinction between regions with short and long ATT is most clearly depicted in Hyb12, whereas in TEnc7, this distinction is less pronounced.

**FIGURE 9:**
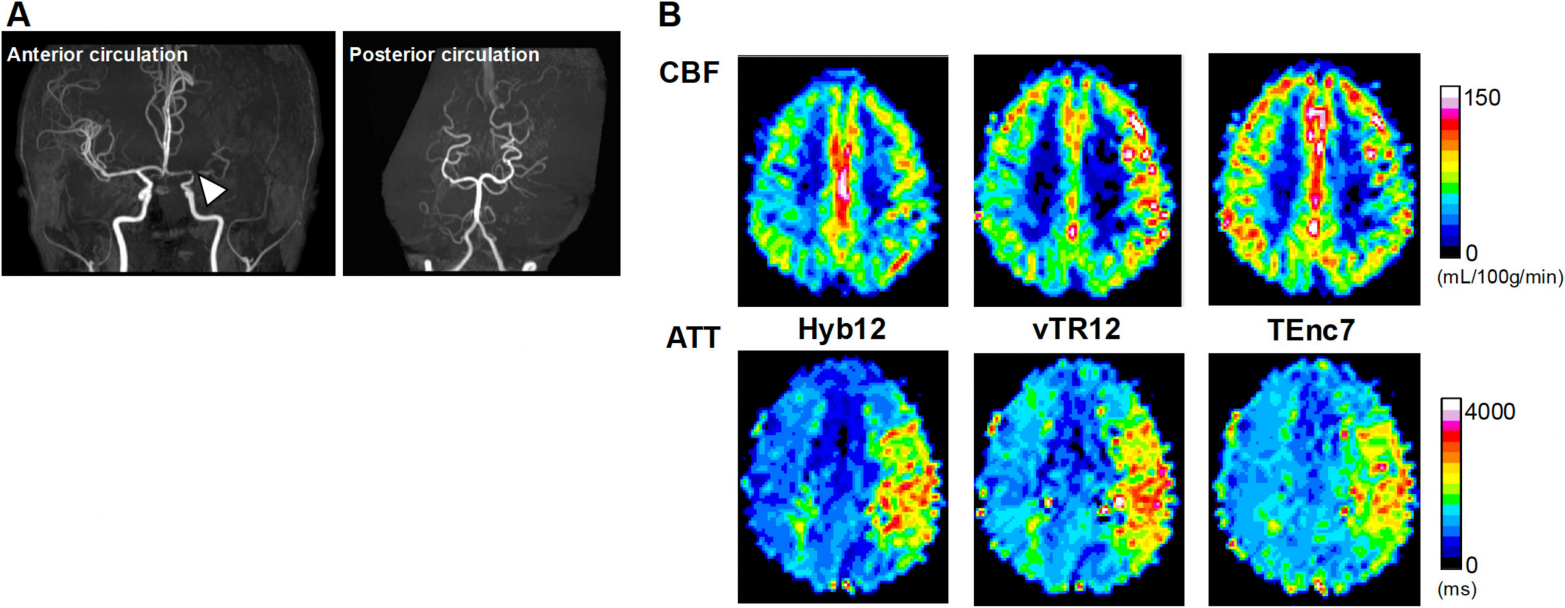
Female in her 30s with left‐sided unilateral moyamoya disease. (A) Three‐dimensional time‐of‐flight MR angiography shows an occlusion around the terminal portion of the left internal carotid artery (arrowhead). (B) CBF maps (top row) show that the CBF observed with Hyb12 is lower than that obtained with the other two schemes. In vTR12, a relative increase in CBF is observed in the left middle cerebral artery territory. (C) ATT maps (bottom row) show prolonged ATT in the left middle cerebral artery territory. Notably, the distinction between regions with short and long ATT is most clearly depicted in Hyb12, whereas in TEnc7, this distinction appears less pronounced.

Figure 5 shows the correlations and Bland–Altman plots for CBF. The CBF measured with Hyb12 shows significant correlations and good agreements with those measured with vTR12 (Figure 5A, *r* = 0.87, *p* < 0.0001, ICC = 0.86) and TEnc7 (Figure 5B, *r* = 0.95, *p* < 0.0001, ICC = 0.94). The Bland–Altman plot analysis for CBF indicates a small fixed bias without significant proportional bias in both comparisons (Figure 5C,D). Figure 6 shows the correlations and Bland–Altman plots for ATT. The ATT measured with Hyb12 shows significant correlations and excellent agreements with those measured with vTR12 (Figure 6A, *r* = 0.97, *p* < 0.0001, ICC = 0.97) and TEnc7 (Figure 6B, *r* = 0.96, *p* < 0.0001, ICC = 0.93). The Bland–Altman plot analysis for ATT between Hyb12 and vTR12 shows a relatively large fixed bias (−234 ms) without significant proportional bias (Figure 6C). In contrast, the analysis for ATT between Hyb12 and TEnc7 reveals a relatively large bias (−162 ms) along with a proportional bias (Figure 6D). Figures 7, 8, 9 show representative cases.

## 4 Discussion

In the present study, as theoretically expected [16], the hybrid scheme provided a higher SNR_t_ than the time‐encoded and variable‐TR schemes. The SNR_t_ of Hyb12 was significantly higher than that of vTR12 and TEnc7 in regions where the ATT was either shorter or longer than its median, and in all vascular territories, including distal regions. The higher SNR_t_ was observed not only in the posterior circulation but also in the anterior circulation, which is more frequently affected in moyamoya disease. Theoretically, time‐encoded PCASL is time‐efficient because information on *N* time points is encoded in *N* + 1 acquisitions, compared to the *N* × 2 acquisitions required for a standard ASL scheme. The combination of signal addition/subtraction over multiple encodings provides noise‐averaging effects in time‐encoded PCASL compared to the two encodings for sequential ASL ^10 23^, which likely resulted in an improved SNR in Hyb12 compared to vTR12. Based on the results from the study by Teeuwisse et al. [23], the SNR will be equal when all scanning time is spent measuring the first block using traditional PCASL or as part of a time‐encoded PCASL experiment with the same total scan time. This implies that, in time‐encoded PCASL, the other timings are essentially obtained “for free.” In contrast, when scanning multiple PLDs with traditional PCASL in variable‐TR scheme while maintaining the same total scan time, fewer averages can be acquired, leading to a lower SNR compared to time‐encoded scheme. However, longer LDs also enhance the SNR_t_, which explains the reason for the lower SNR_t_ of TEnc7 compared to Hyb12. Our results prove the concept that the use of time‐encoded PCASL in the hybrid scheme overcomes the lower SNR issue of variable‐TR PCASL, whereas still enough flexibility in timing scan be achieved to allow accurate quantification. This concept behind the hybrid approach is especially advantageous in this disease where the ATT can be prolonged and the magnetization of the labeled blood decays significantly before it reaches the tissue. Therefore, the sequence is crucially dependent on both sampling over a wide range of PLDs and sufficient SNR.

Significant differences in CBF and ATT measurements were observed between Hyb12 and the two reference sequences: CBF was lower, and ATT was shorter when measured with Hyb12 compared to the other two methods. Several possible explanations could account for these differences. Previous simulation studies have demonstrated that reduced SNR results in an overestimation of CBF, whereas increased SNR leads to more accurate CBF measurements [24, 25]. The higher SNR obtained with Hyb12 should be advantageous for accurate quantification. The interruption of labeling by the insertion of background suppression pulses in the variable‐TR scheme has caused some additional loss of labeling. On the other hand, TEnc7 may not have accurately assessed the early or late arrival of labeled blood due to the smaller number of time points than Hyb12, although also vTR12 showed similar trends and this sequence has similar timings as Hyb12.

The relative CBF showed no notable underestimation or overestimation with Hyb12. However, the relative CBF obtained with vTR12 was higher than that of the other two schemes in the middle and distal portions of the MCA territories and lower in the proximal and middle portions of the PCA territories. The reasons for these differences remain unclear. One possible explanation is that in the variable TR scheme, labeling is interrupted to insert background suppression pulses, which may lead to a reduction in labeling efficiency, as previously mentioned. In actual measurements (Figure 2C), vTR12 exhibits the lowest SNR in regions with prolonged ATT. Consequently, insufficient SNR in regions with delayed ATT may lead to an overestimation of CBF. These tendencies are illustrated in Figures 7, 8, 9. Labeled proton spins in arteries that have not yet reached the brain tissue might be one of the reasons.

CBF and ATT measured with Hyb12 show excellent correlations and good to excellent agreement with those measured with the other two schemes, although small biases were observed as described in the previous paragraph. These results confirm the quantitative capability of the hybrid scheme for perfusion imaging.

A recent study also confirms that a longer LD of 3 s further improves the SNR especially in the regions with prolonged ATT in this disease [26]. The longer LD helps both in increasing SNR and in increasing blood volume labeled by the long label may have allowed for a more accurate measurement of ATT, as the label persisted even in tissues with delayed blood arrival. Another study demonstrated the utility of a combined acquisition of time‐encoded PCASL and long‐labeled (LD = 4 s), long‐delayed (PLD = 3 s) PCASL (LLLD‐PCASL) in moyamoya disease [27]. Alternatively, the recently proposed VESPA method [28], combining velocity and spatially selective ASL with time‐encoded PCASL, achieves accurate CBF even when ATT is long. In the future comparisons between these schemes, taking into account the needs of clinicians would be useful.

The present study has several limitations. First, there was no real gold standard for quantitative perfusion parameters such as those obtained by SPECT or PET. Because the quantitative performance of the variable‐TR scheme in this disease has been demonstrated by comparison with SPECT [13], we considered it as our reference in this study. Second, the spatial resolution of ASL was not high (4.0 × 4.0 × 8.0 mm) compared to dynamic susceptibility contrast perfusion imaging. However, this resolution is comparable to that of brain perfusion SPECT or PET (typically 4–6 mm) [29]. Third, the number of patients (*N* = 20) was small. Further validation of the variable TR scheme should be performed in a larger series of patients. Fourth, in this study, we measured the SNR using the three highest signals voxel by voxel. However, the SNR defined here may not directly correspond to the precision of CBF calculation. Future studies are needed to evaluate CBF quantification in terms of the SNR measurement method. Finally, we did not control for patients' conditions at the time of imaging. Scan duration, awake state, time of day, and use of sedation may have influenced the perfusion parameters, although the three sequences were acquired in a single session, and therefore, it can be expected that the condition of the patients did not change much between approaches.

## 5 Conclusion

The hybrid scheme provided a higher SNR_t_ than the time‐encoded and variable‐TR PCASL schemes. This is advantageous for better fitting in kinetic models, especially for regions with delayed transit times frequently encountered in moyamoya disease. The quantitative capability of the hybrid scheme for CBF and ATT for CBF and ATT was confirmed in this disease by the comparisons with the two other schemes. The hybrid scheme combines the advantages of high SNR with the flexibility in choice of LD/PLD combinations as needed to accurately assess cerebral perfusion in the presence of prolonged ATT, as seen in moyamoya disease.

## Data Availability

The data that support the findings of this study are available on request from the corresponding author. The data are not publicly available due to privacy or ethical restrictions.

## References

[1] J. Suzuki and A. Takaku , “Cerebrovascular “Moyamoya” Disease. Disease Showing Abnormal Net‐Like Vessels in Base of Brain,” Archives of Neurology 20, no. 3 (1969): 288–299, 10.1001/archneur.1969.00480090076012. 5775283

[2] K. Ikezaki , T. Matsushima , Y. Kuwabara , S. O. Suzuki , T. Nomura , and M. Fukui , “Cerebral Circulation and Oxygen Metabolism in Childhood Moyamoya Disease: A Perioperative Positron Emission Tomography Study,” Journal of Neurosurgery 81, no. 6 (1994): 843–850, 10.3171/jns.1994.81.6.0843. 7965114

[3] Y. Kuwabara , Y. Ichiya , M. Sasaki , et al., “Cerebral Hemodynamics and Metabolism in Moyamoya Disease—A Positron Emission Tomography Study,” Clinical Neurology and Neurosurgery Oct 1997 99, no. Suppl 2 (1997): S74–S78, 10.1016/s0303-8467(97)00061-9. 9409411

[4] T. Tominaga , N. Suzuki , S. Miyamoto , et al., “Recommendations for the Management of Moyamoya Disease: A Statement From Research Committee on Spontaneous Occlusion of the Circle of Willis (Moyamoya Disease) [2nd Edition],” Surgery for Cerebral Stroke 46, no. 1 (2018): 1–24, 10.2335/scs.46.1.

[5] M. Fujimura , T. Tominaga , S. Kuroda , et al., “2021 Japanese Guidelines for the Management of Moyamoya Disease: Guidelines from the Research Committee on Moyamoya Disease and Japan Stroke Society,” Neurologia Medico‐Chirurgica 62, no. 4 (2022): 165–170, 10.2176/jns-nmc.2021-0382. 35197402 PMC9093674

[6] D. C. Alsop , J. A. Detre , X. Golay , et al., “Recommended Implementation of Arterial Spin‐Labeled Perfusion MRI for Clinical Applications: A Consensus of the ISMRM Perfusion Study Group and the European Consortium for ASL in Dementia,” Magnetic Resonance in Medicine 73, no. 1 (2015): 102–116, 10.1002/mrm.25197. 24715426 PMC4190138

[7] K. Setta , T. Matsuda , M. Sasaki , et al., “Diagnostic Accuracy of Screening Arterial Spin‐Labeling MRI Using Hadamard Encoding for the Detection of Reduced CBF in Adult Patients with Ischemic Moyamoya Disease,” AJNR. American Journal of Neuroradiology 42, no. 8 (2021): 1403–1409, 10.3174/ajnr.A7167. 34016589 PMC8367606

[8] W. Dai , A. Shankaranarayanan , and D. C. Alsop , “Volumetric Measurement of Perfusion and Arterial Transit Delay Using Hadamard Encoded Continuous Arterial Spin Labeling,” Magnetic Resonance in Medicine 69, no. 4 (2013): 1014–1022, 10.1002/mrm.24335. 22618894 PMC3427721

[9] J. A. Wells , M. F. Lythgoe , D. G. Gadian , R. J. Ordidge , and D. L. Thomas , “In Vivo Hadamard Encoded Continuous Arterial Spin Labeling (H‐CASL),” Magnetic Resonance in Medicine 63, no. 4 (2010): 1111–1118, 10.1002/mrm.22266. 20373414

[10] M. J. van Osch , W. M. Teeuwisse , Z. Chen , Y. Suzuki , M. Helle , and S. Schmid , “Advances in Arterial Spin Labeling MRI Methods for Measuring Perfusion and Collateral Flow,” Journal of Cerebral Blood Flow and Metabolism: Official Journal of the International Society of Cerebral Blood Flow and Metabolism 38, no. 9 (2018): 1461–1480, 10.1177/0271678X17713434. 28598243 PMC6120125

[11] M. E. Johnston , K. Lu , J. A. Maldjian , and Y. Jung , “Multi‐TI Arterial Spin Labeling MRI With Variable TR and Bolus Duration for Cerebral Blood Flow and Arterial Transit Time Mapping,” IEEE Transactions on Medical Imaging 34, no. 6 (2015): 1392–1402, 10.1109/TMI.2015.2395257. 25616010

[12] M. Obara , O. Togao , T. Wada , et al., “Pseudo‐Continuous Arterial Spin Labeling Using Multiple Label‐ and Post‐Label Duration With Dynamically Optimized Background Suppression,” Proceedings on International Society for Magnetic Resonance in Medicine 29 (2021): 870.

[13] O. Togao , M. Obara , K. Yamashita , et al., “Assessment of Cerebral Perfusion in Moyamoya Disease With Dynamic pseudo‐Continuous Arterial Spin Labeling Using a Variable Repetition Time Scheme With Optimized Background Suppression,” Neuroradiology 65, no. 3 (2023): 529–538, 10.1007/s00234-022-03095-5. 36434310

[14] M. Obara , O. Togao , L. Vaclavu , et al., “Comparison of a Hybrid Multi‐Delay Pseudo‐Continuous Arterial Spin Labelling Scheme with Time‐Encoded and Variable‐TR Schemes,” Proceedings on International Society for Magnetic Resonance in Medicine 31 (2023): 3392.

[15] J. G. Woods , M. A. Chappell , and T. W. Okell , “Designing and Comparing Optimized pseudo‐Continuous Arterial Spin Labeling Protocols for Measurement of Cerebral Blood Flow,” NeuroImage 223 (2020): 117246, 10.1016/j.neuroimage.2020.117246. 32853814 PMC7762814

[16] J. G. Woods , E. Achten , I. Asllani , et al., “Recommendations for Quantitative Cerebral Perfusion MRI Using Multi‐Timepoint Arterial Spin Labeling: Acquisition, Quantification, and Clinical Applications,” Magnetic Resonance in Medicine 92, no. 2 (2024): 469–495, 10.1002/mrm.30091. 38594906 PMC11142882

[17] M. Obara , M. van Cauteren , M. Honda , Y. Imai , and K. Kuroda , “Assessment of Improved Motion‐Sensitized Driven Equilibrium (iMSDE) for Multi‐Contrast Vessel Wall Screening,” Magnetic Resonance in Medical Sciences 13, no. 2 (2014): 139–144, 10.2463/mrms.2013-0036. 24769630

[18] R. B. Buxton , L. R. Frank , E. C. Wong , B. Siewert , S. Warach , and R. R. Edelman , “A General Kinetic Model for Quantitative Perfusion Imaging With Arterial Spin Labeling,” Magnetic Resonance in Medicine 40, no. 3 (1998): 383–396, 10.1002/mrm.1910400308. 9727941

[19] Q. Qin , A. J. Huang , J. Hua , J. E. Desmond , R. D. Stevens , and P. C. van Zijl , “Three‐Dimensional Whole‐Brain Perfusion Quantification Using pseudo‐Continuous Arterial Spin Labeling MRI at Multiple Post‐Labeling Delays: Accounting for Both Arterial Transit Time and Impulse Response Function,” NMR in Biomedicine 27, no. 2 (2014): 116–128, 10.1002/nbm.3040. 24307572 PMC3947417

[20] J. Ashburner and K. J. Friston , “Voxel‐Based Morphometry—The Methods,” NeuroImage 11, no. 6 (2000): 805–821, 10.1006/nimg.2000.0582. 10860804

[21] H. J. Mutsaerts , “ATT Based Flow Territories,” Figshare. Dataset, 10.6084/m9.figshare.1488674.v1.

[22] T. K. Koo and M. Y. Li , “A Guideline of Selecting and Reporting Intraclass Correlation Coefficients for Reliability Research,” Journal of Chiropractic Medicine 15, no. 2 (2016): 155–163, 10.1016/j.jcm.2016.02.012. 27330520 PMC4913118

[23] W. M. Teeuwisse , S. Schmid , E. Ghariq , I. M. Veer , and M. J. van Osch , “Time‐Encoded Pseudocontinuous Arterial Spin Labeling: Basic Properties and Timing Strategies for Human Applications,” Magnetic Resonance in Medicine 72, no. 6 (2014): 1712–1722, 10.1002/mrm.25083. 24395462

[24] J. A. Wells , D. L. Thomas , M. D. King , A. Connelly , M. F. Lythgoe , and F. Calamante , “Reduction of Errors in ASL Cerebral Perfusion and Arterial Transit Time Maps Using Image De‐Noising,” Magnetic Resonance in Medicine 64, no. 3 (2010): 715–724, 10.1002/mrm.22319. 20578044

[25] J. Guo , S. J. Holdsworth , A. P. Fan , et al., “Comparing Accuracy and Reproducibility of Sequential and Hadamard‐Encoded Multidelay Pseudocontinuous Arterial Spin Labeling for Measuring Cerebral Blood Flow and Arterial Transit Time in Healthy Subjects: A Simulation and In Vivo Study,” Journal of Magnetic Resonance Imaging 47, no. 4 (2018): 1119–1132, 10.1002/jmri.25834. 28792653 PMC5807238

[26] O. Togao , M. Obara , R. Mikayama , et al., “The Effect of Long Label Duration on Hybrid Multi‐Delay PCASL of Time‐Encoded and Variable‐TR Schemes for the Assessment of Cerebral Perfusion in Moyamoya Disease,” Proceedings of the International Society for Magnetic Resonance in Medicine 32 (2024): 2327.

[27] K. Takata , H. Kimura , S. Ishida , et al., “Assessment of Arterial Transit Time and Cerebrovascular Reactivity in Moyamoya Disease by Simultaneous PET/MRI,” Diagnostics (Basel) 13, no. 4 (2023): 756, 10.3390/diagnostics13040756. 36832244 PMC9955140

[28] J. G. Woods , E. C. Wong , E. C. Boyd , and D. S. Bolar , “VESPA ASL: VElocity and SPAtially Selective Arterial Spin Labeling,” Magnetic Resonance in Medicine 87, no. 6 (2022): 2667–2684, 10.1002/mrm.29159. 35061920

[29] M. Wintermark , M. Sesay , E. Barbier , et al., “Comparative Overview of Brain Perfusion Imaging Techniques,” Journal of Neuroradiology 32, no. 5 (2005): 294–314, 10.1016/s0150-9861(05)83159-1. 16424829

